# A multicenter study of asymmetric and symmetric dimethylarginine as predictors of mortality risk in hospitalized COVID-19 patients

**DOI:** 10.1038/s41598-024-66288-3

**Published:** 2024-07-08

**Authors:** Juliane Hannemann, Anne Zink, Yoana Mileva, Paul Balfanz, Edgar Dahl, Sonja Volland, Thomas Illig, Edzard Schwedhelm, Florian Kurth, Alexandra Stege, Martin Aepfelbacher, Armin Hoffmann, Rainer Böger

**Affiliations:** 1 Institute of Clinical Pharmacology and Toxicology, University Medical Center Hamburg-Eppendorf, Hamburg, Germany; 2 Department of Cardiology, Angiology and Intensive Care Medicine, Medical Clinic I, University Hospital Aachen, Aachen, Germany; 3 Department of Physiology, Henri Mondor Hospital, FHU-SENEC, INSERM U955, Université Paris-Est Créteil (UPEC), AP-HP, Créteil, France; 4 Institute of Pathology and Central Biobank, University Hospital Aachen, Aachen, Germany; 5 Hannover Unified Biobank, Medizinische Hochschule Hannover, Hannover, Germany; 6 German Centre for Cardiovascular Research (DZHK), partner site Hamburg/Kiel/Lübeck, Hamburg, Germany; 7 Department of Infectious Diseases and Critical Care Medicine, Charité Universitätsmedizin Berlin, Berlin, Germany; 8 Central Biobank Charité, Charité Universitätsmedizin Berlin, Berlin, Germany; 9 Institute of Medical Microbiology, Virology and Hygiene, University Medical Center Hamburg-Eppendorf, Hamburg, Germany

**Keywords:** Infectious diseases, Nitric oxide, Intensive care, Biomarker, Infectious diseases, Biomarkers

## Abstract

Mortality of patients hospitalized with COVID-19 has remained high during the consecutive SARS-CoV-2 pandemic waves. Early discrimination of patients at high mortality risk is crucial for optimal patient care. Symmetric (SDMA) and asymmetric dimethylarginine (ADMA) have been proposed as possible biomarkers to improve risk prediction of COVID-19 patients. We measured SDMA, ADMA, and other L-arginine-related metabolites in 180 patients admitted with COVID-19 in four German university hospitals as compared to 127 healthy controls. Patients were treated according to accepted clinical guidelines and followed-up until death or hospital discharge. Classical inflammatory markers (leukocytes, CRP, PCT), renal function (eGFR), and clinical scores (SOFA) were taken from hospital records. In a small subgroup of 23 COVID-19 patients, sequential blood samples were available and analyzed for biomarker trends over time until 14 days after admission. Patients had significantly elevated SDMA, ADMA, and L-ornithine and lower L-citrulline concentrations than controls. Within COVID-19 patients, SDMA and ADMA were significantly higher in non-survivors (n = 41, 22.8%) than in survivors. In ROC analysis, the optimal cut-off to discriminate non-survivors from survivors was 0.579 µmol/L for SDMA and 0.599 µmol/L for ADMA (both *p* < 0.001). High SDMA and ADMA were associated with odds ratios for death of 11.45 (3.37–38.87) and 5.95 (2.63–13.45), respectively. Analysis of SDMA and ADMA allowed discrimination of a high-risk (mortality, 43.7%), medium-risk (15.1%), and low-risk group (3.6%); risk prediction was significantly improved over classical laboratory markers. We conclude that analysis of ADMA and SDMA after hospital admission significantly improves risk prediction in COVID-19.

## Introduction

Infection with the newly emerged SARS-CoV2 has afflicted millions of people around the globe during the COVID-19 pandemic that lasted from March 2020 to May 2023. This pandemic evolved with several major waves of infection, during which the pathogenicity of the virus underwent several changes over time due to mutations in the virus genome, as reviewed by Nugent^1^. The COVID-19 disease took a mild to moderate course in the majority of those infected; however, some patients were hospitalized due to severe symptoms and a comparatively high proportion of hospitalized patients did not survive their disease. The pandemic caused an estimated 18 million deaths worldwide, resulting in a significant excess mortality.

In total, 765 million people worldwide were affected by COVID-19, with an overall mortality rate of 0.9%^2^. However, the mortality rate of those who suffered from severe COVID-19 requiring hospitalization varied during the three major pandemic waves, reaching about 40–50% in early 2020, about 11% in late 2020, then rising again to around 21% in early 2021 and declining thereafter^3^.

As routine clinical scores and biomarkers only insufficiently allowed to discriminate between patients at high and low risk of in-hospital mortality, many investigators took efforts to find biomarkers that have better prognostic value when taken early after hospital admission^4–6^. The nitric oxide (NO) pathway is a major regulator of vascular function and immune defense in severe infections and sepsis, mediated by the endothelial and inducible isoforms of NO synthase^7^. We have previously published data showing that the circulating levels of asymmetric dimethylarginine (ADMA) and symmetric dimethylarginine (SDMA), two methylated variants of the amino acid L-arginine which acts as the natural substrate for enzymatic NO production, are suitable biomarkers for disease severity and outcome in sepsis patients^8,9^. ADMA directly interferes with NO synthesis by competing with L-arginine for binding to the enzyme’s active site^10^, whilst both dimethylarginines reduce cellular bioavailability of L-arginine through inhibition of cellular L-arginine transport proteins^11^. In addition, arginase is another enzyme that consumes L-arginine as a substrate by converting it into L-ornithine, thereby potentially reducing substrate availability for NO synthesis^12^. Arginase has been shown to be upregulated in sickle cell disease, type 2 diabetes mellitus diseases, and other conditions where it limits NO-mediated vascular relaxation^13,14^.

In a pilot study of 33 hospitalized patients during the first pandemic wave, we found that sequential analysis of ADMA and SDMA in blood samples taken on the first day of hospitalization for COVID-19 significantly improved the ability to predict in-hospital mortality as compared to traditional risk scores and biomarkers like SOFA, leukocyte count, C-reactive protein, and pro-calcitonin^15^. Subsequently, other investigators corroborated our findings with different analytical techniques^16–18^, but also in small patient cohorts. In addition, in a collaborative study with Italian researchers, we recently reported that severely affected COVID-19 patients with low pulmonary vasodilation detected by chest CT scan could be identified by high plasma ADMA concentration; these patients had a poor outcome^19^. By contrast, COVID-19 patients with severe clinical disease but with high pulmonary vasodilation showed ADMA levels comparable to those with mild disease as well as better outcome. This data suggests that there might be a pathophysiological role of dimethylarginines in controlling pulmonary blood flow during SARS-CoV2 infection; their addition to the clinical laboratory marker spectrum might enhance identification of high-risk COVID-19 patients. In the present study, we investigated the roles of ADMA and SDMA as predictive biomarkers of COVID-19-related in-hospital mortality in a multicenter study involving four University medical centers in Germany and a large number of COVID-19 patients. In addition, we analyzed a broader spectrum of L-arginine-related metabolites including L-ornithine, the product of arginases, and L-citrulline, the product of the NO synthesis pathway, in order to receive a more complete picture of the disturbances in L-arginine biochemical pathways.

## Patients and methods/material and methods

### Study participants

**Figure 1:**
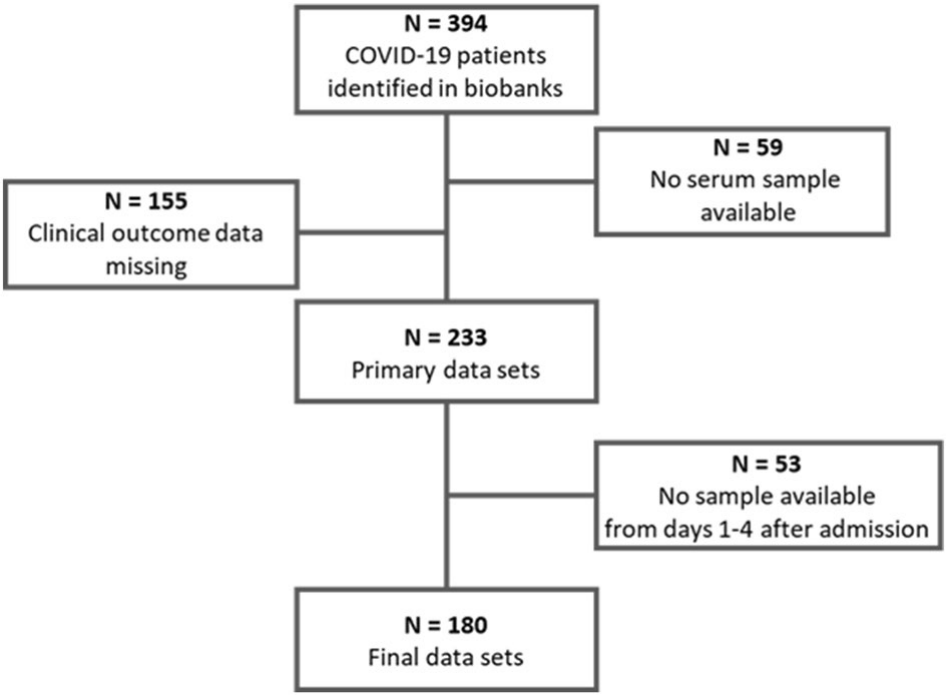
CONSORT flow diagram of our study.

We identified 394 patients with COVID-19 who were included in one of the four participating biobanks. After exclusion of patients with missing serum samples (N = 59), patients with missing outcome data (N = 155), and patients for whom no serum sample was available from days 1–4 after hospital admission (N = 53), we included 180 patients who were admitted with symptomatic COVID-19 to one of the four participating medical centers between January 02, 2020 and January 22, 2021 (Fig. 1). Patients were included if the main cause for hospitalization was COVID-19; all patients had a positive SARS-CoV-2 PCR test result in respiratory samples analyzed on admission or externally prior to admission. Participating centers were the University Medical Center Hamburg-Eppendorf (UKE), the University Clinic Aachen (UKA), the Medical School of Hannover (MHH), and the Charité Berlin (ChB).

As a control group, we recruited 127 healthy age- and sex-matched blood donors who consented to donate the remainder of a blood sample that was routinely collected for clinical chemistry testing for research purposes. All blood donors had been evaluated for the absence of diseases that prevented them from donating blood, and had consented to participation after written and verbal information.

### Study protocol

The entire study protocol was approved by the Ethics Committee of the Hamburg Board of Physicians (2021-100662-BO-ff and 2022-300225-WF). All patients gave their informed consent for their blood samples to be included into the local biobanks. The Ethics Committee of the Medical Faculty of RWTH Aachen had consented the Covid-19 Aachen study (COVAS) according to its vote EK080/20 and the regulations of the RWTH centralized Biomaterial Bank (RWTH cBMB; vote EK206/09). The Ethics Committee of the Hannover Medical School (MHH) had approved the collection of biomaterial and data from COVID-19 patients and the broad molecular characterization of the MWK COVID-19 cohort of the Hannover Unified biobank of MHH (vote number 9001_BO_K). Patients at Chb were included as part of the Pa-Covid-19 study (DRKS00021688) which was approved by the Charité Ethics Committee (EA2/066/20)^20^. All investigations were performed in accordance with the Declaration of Helsinki in its latest revision. No further patient selection criteria were applied. Serum samples had been collected from all patients and stored in the local biobank frozen at -80°C until analysis of biomarkers. Samples from days 1–4 after admission were available from all patients; in addition, consecutive samples taken during up to 14 days after admission were available from a subgroup of 23 patients. Routine clinical data, laboratory parameters, and parameters of clinical disease course and outcome were collected locally and transmitted for statistical analysis in a pseudonymized manner. All patients were treated according to best medical practice and individual clinical needs. Patients were either isolated under standard care or treated in the intensive care units (ICU). The decision on treatment strategies was based on clinical judgment of the severity of the disease and the presence or absence of acute respiratory distress syndrome (ARDS). Follow-up was continued for all patients until in-hospital death or discharge.

### Assessment of biomarkers by UPLC-MS/MS

Validated protocols for liquid chromatography-tandem mass spectrometry (LC–MS/MS) were used to quantify L-arginine, L-ornithine, L-citrulline, ADMA and SDMA concentrations in serum^21^. Briefly, 25 μL of serum were diluted in methanol to which stable isotope labelled internal standards had been added. Subsequently, the compounds were converted into their butyl ester derivatives and quantified by LC–MS/MS (Xevo TQ-S cronos, Waters GmbH, Eschborn, Germany). Compounds were separated on an Aquity UPLC BEH C18 column (2·1 × 50 mm, 1.7 μm, Waters GmbH). The coefficient of variation for the quality control samples was below 15% for all compounds. All biomarkers were measured by investigators blinded to patient outcome.

### Assessment of clinical patient status

Patients were assessed as eligible based on a positive RT-PCR test for SARS-CoV-2 in a respiratory tract sample as previously described^22^. Vital parameters presented in this study were taken between four and 24 h following hospital admission or intubation. The SOFA score was recorded as a measure of clinical patient status^23^. Acute respiratory distress syndrome (ARDS) was diagnosed according to the Berlin definition^24^. Acute kidney injury was defined according to the AKIN criteria^25^ and/or need for continuous veno-venous hemofiltration in patients with no pre-existing chronic renal failure. Patients with a body mass index (BMI) of 25 to < 30 kg/m^2^ were classified as overweight and those with BMI ≥ 30 kg/m^2^ as obese. Diabetes and prediabetes were defined by clinical history, medication and HbA1c values ≥ 6.5% or ≥ 5.7 to < 6.5%, respectively. Serum and whole blood samples were obtained routinely at the time of admission. Complete blood count, coagulation tests, inflammatory markers [circulating levels of C-reactive protein (CRP), pro-calcitonin (PCT)] and creatinine levels in blood were measured among other tests. Creatinine clearance was estimated using the CKD-EPI formula^26^.

### Statistical analyses

All variables were tested for normal distribution using the Kolmogorov–Smirnov test. Data are presented as mean with standard deviation (SD). Differences between groups were tested for significance using the nonparametric Mann–Whitney U test for two groups or the Kruskal–Wallis analysis of variance for more than two groups. The Chi^2^ test was used for comparison of categorical variables between groups. Time courses of ADMA and SDMA concentrations were examined using repeated measures two-way ANOVA followed by Tukey’s multiple comparisons test. Spearman’s rank correlation was used to assess pairwise correlations. Survival analyses were performed using Kaplan–Meier curves comparing patients with ADMA and SDMA above or below the cut-off value determined in receiver-operated curve (ROC) analyses. The Youden index was calculated to identify the optimal cut-off for biomarkers^27^. Hazard ratios (HR) and 95% confidence intervals (CI) were calculated by multivariable-adjusted logistic regression analyses. As we had identified two biomarkers, ADMA and SDMA, as predictors of COVID-19 mortality, we analyzed additional models using (SDMA + ADMA) or (SDMA × ADMA) as variables, respectively. In addition, we performed a decision tree analysis to determine risk upon sequential analysis of SDMA and ADMA. Cut-offs to separate risk groups were based on values determined in ROC analysis for both biomarkers. All statistical analyses were performed using SPSS (version 25; IBM Corporation, Armonk, NY, USA) and GraphPad Prism (version 6.01, GraphPad Software, San Diego, CA, USA). For all tests, *p* < 0.05 was considered statistically significant.

## Results

### Baseline characteristics

Patients had a mean age of 62.1 ± 15.4 years (range 22–91 years); 64/180 patients (35.6%) were female. Hospital admission was on day 6.7 ± 6.2 after the onset of COVID-19 symptoms. Baseline patient characteristics are shown in Table 1 for the entire cohort and for patients who survived or died during hospitalization. The first blood sample was taken on days 1–4 after hospital admission (mean, day 2.0 ± 1.2).

**Table 1:** Baseline characteristics of the study cohort by survival.

	Total	Survived	Deceased	*p*
Number of patients	180	139	41	n.a
Females, N (%)	64 (35.6)	51 (36.7)	13 (31.7)	0.552
Age, years	62.1 ± 15.4	60.6 ± 16.0	67.1 ± 11.9	0.017
BMI, kg/m^2^	28.8 ± 7.7	28.1 ± 6.5	32.2 ± 11.9	0.124
Days after symptom onset	6.7 ± 6.2	6.9 ± 5.7	5.9 ± 7.6	0.463
Sys blood pressure, mm Hg	126.8 ± 21.9	130.0 ± 20.8	118.0 ± 22.6	0.011
Dia blood pressure, mm Hg	73.4 ± 13.7	75.6 ± 13.4	67.3 ± 12.8	0.005
Heart rate, 1/min	87.8 ± 19.8	86.3 ± 17.6	91.8 ± 24.9	0.199
SOFA score	3.3 ± 3.8	1.9 ± 2.6	6.7 ± 4.4	< 0.001
SaO_2_, %	93.8 ± 5.5	94.7 ± 4.4	91.2 ± 7.4	< 0.001
Cardiovascular disease	27 (15.0)	21 (15.1)	6 (14.6)	1.000
Chronic lung disease	30 (16.7)	19 (13.7)	11 (26.8)	0.035
Cancer	46 (25.6)	37 (26.6)	9 (22.0)	0.511
Chronic kidney disease	37 (20.6)	32 (23.0)	5 (12.2)	0.062
Diabetes mellitus	12 (20.4)	7 (5.0)	5 (12.2)	0.126
Hypertension	30 (16.7)	23 (16.5)	7 (17.1)	1.000
Current smoker	43 (23.8)	29 (20.9)	14 (33.3)	0.079
Leukocyte count, 1/nL	9.2 ± 6.5	8.5 ± 6.2	11.6 ± 6.8	0.069
C-reactive protein, mg/L	101.3 ± 98.8	75.3 ± 72.2	188.0 ± 122.3	< 0.001
Pro-calcitonin, ng/mL	2.7 ± 14.2	0.2 ± 0.4	11.1 ± 27.9	0.018
D-dimers, ng/mL	2074 ± 4910	884 ± 1464	4964 ± 8202	< 0.001
Creatinine, mg/dL	1.07 ± 0.57	0.99 ± 0.44	1.36 ± 0.84	0.041
Hospital treatment, days	17.9 ± 19.4	17.8 ± 21.7	26.4 ± 14.3	0.018
ARDS, N (%)	81 (45.0)	52 (37.4)	29 (70.7)	< 0.0001
ICU treatment, N (%)	81 (45.0)	52 (37.4)	29 (70.7)	< 0.0001
Mech. ventilation, N (%)	51 (28.3)	24 (17.3)	27 (65.9)	< 0.0001
ECMO treatment, N (%)	35 (19.4)	15 (10.8)	20 (48.8)	< 0.0001
Deceased, N (%)	41 (22.8)	n.a	n.a	n.a

Data are mean ± standard deviation if not indicated otherwise.

ARDS, acute respiratory distress syndrome; BMI, body mass index; ECMO, extracorporeal membrane oxygenation; ICU, intensive care unit; SOFA score, sepsis-related organ failure assessment score; SaO_2_, arterial oxygen saturation.

Mean age of controls was 54.9 ± 11.9 years (range 22–74 years) and 44/127 (34.6%) of controls were women. According to their status as blood donors, none of the control subjects had major chronic diseases, nor fever or any acute signs of infection; their routine laboratory analyses showed no pathological findings. We were unable to include healthy controls for COVID-19 patients older than 74 years, as individuals in the age range above that age were not present amongst blood donors. Thus, matching of COVID-19 patients and health controls for age was incomplete.

### Clinical course of the patients

The mean duration of hospitalization was 19.7 ± 20.6 days. 41 patients (22.8%) died during hospitalization. 81 patients (45.0%) were classified as having acute respiratory distress syndrome (ARDS), and were treated in ICU. 51 patients (28.3%) received mechanical ventilation, and 35 patients (19.4%) were treated with ECMO. The rates of ARDS and subsequent ICU treatment, mechanical ventilation, and ECMO therapy were significantly higher in the subgroup of patients who died than in survivors (Table 1). Survivors had lower SOFA scores at admission; also, inflammatory laboratory markers like leukocyte count, CRP, and pro-calcitonin taken at the same time as L-arginine-related biomarkers were significantly higher in patients who died than in survivors (Table 1).

### Concentrations of L-arginine-associated biomarkers in COVID-19 patients versus controls

**Figure 2:**
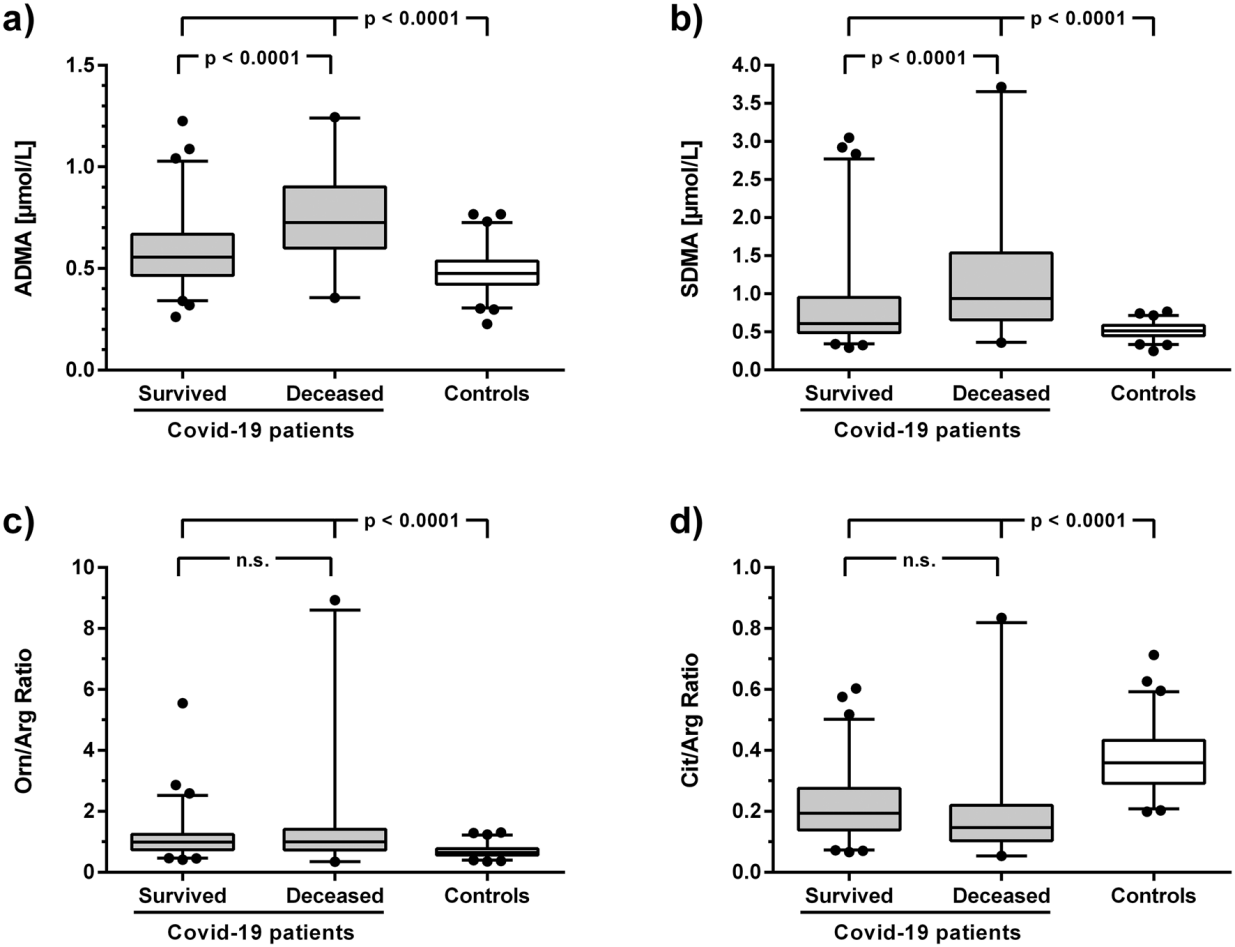
Box plots showing the serum concentrations of ADMA (**a**), SDMA (**b**), the L-ornithine/L-arginine ratio (**c**), and the L-citrulline/L-arginine ratio (**d**) in hospitalized Covid-19 patients who survived or died during hospitalization, as compared to age- and sex-matched healthy controls. Boxes show the median and interquartile range of the data, with whiskers representing the 2.5th to 97.5th percentiles; data points outside of this distribution are plotted individually. Statistical significances were calculated by one-way ANOVA followed by Tukey's multiple comparisons test. ADMA, asymmetric dimethylarginine; Cit/Arg Ratio, L-citrulline/L-arginine ratio; Orn/Arg Ratio, L-ornithine/L-arginine ratio; SDMA, symmetric dimethylarginine.

The concentrations of ADMA and SDMA in the first available serum sample after hospital admission were significantly higher in COVID-19 patients than in healthy blood donors (Fig. 2a,b). The serum concentration of L-ornithine was significantly higher and that of L-citrulline was significantly lower in COVID-19 patients than in controls, whilst there was no significant difference in L-arginine concentration (Table 2, supplementary Figure 1). These differences in biomarker concentrations resulted in a significant elevation of the L-ornithine/L-arginine ratio (Orn/Arg Ratio), whilst the L-citrulline/L-arginine ratio (Cit/Arg Ratio) was significantly reduced in COVID-19 patients versus controls (Fig. 2c,d). When we restricted our analysis to fully matched COVID-19 patients and controls, the differences in mean metabolite concentrations remained unchanged (Supplementary Table 1).

**Table 2:** Biomarker concentrations at baseline by survival.

	Covid-19 patients	Controls	*p*	Covid-19 patients		
				Survived	Deceased	*p*
N	180	127		139	41	n.a
L-Arginine	112.8 ± 42.3	100.3 ± 25.7	n.s	109.6 ± 40.1	123.9 ± 48.0	n.s
L-Ornithine	109.7 ± 37.5	66.7 ± 18.4	**< 0.0001**	106.4 ± 35.7	121.0 ± 41.8	**0.027**
L-Citrulline	21.5 ± 10.1	35.6 ± 8.7	**< 0.0001**	22.1 ± 10.3	19.6 ± 9.4	n.s
ADMA	0.625 ± 0.189	0.480 ± 0.098	**< 0.0001**	0.590 ± 0.168	0.743 ± 0.209	**< 0.0001**
SDMA	0.874 ± 0.572	0.519 ± 0.094	**< 0.0001**	0.795 ± 0.526	1.142 ± 0.643	**< 0.0001**

Significant values are in bold.

Data are mean ± standard deviation. Concentrations are given as µmol/L.

ADMA, asymmetric dimethylarginine; n.a., not assessed; n.s., not significant; SDMA, symmetric dimethylarginine. *P* values are from oneway ANOVA with adjustment of p for multiple testing.

Within the group of COVID-19 patients, ADMA and SDMA concentrations were significantly higher in patients who died during hospitalization than in survivors (Fig. 2a,b), as was L-ornithine concentration, whereas there were no significant differences in L-arginine and L-citrulline concentrations (Table 2). The Orn/Arg Ratio and the Cit/Arg Ratio showed no significant differences between survivors and non-survivors (Fig. 2c,d).

### Associations of ADMA and SDMA with mortality in COVID-19

In univariate logistic regression analyses, ADMA and SDMA were significantly associated with survival (ADMA, OR 63.051 (9.122–435.821), *p* < 0.001; SDMA; OR 2.514 (1.410–4.480), *p* = 0.002). Both biomarkers remained significantly associated with mortality in a model adjusted for age and sex and in a model adjusted for age, sex, and leukocyte count, whilst only ADMA retained statistical significance in a models adjusted for age, sex, and CRP. Both biomarkers showed no significant associations in models adjusted for age, sex, and eGFR or PCT (Table 3).

**Table 3:** Multivariable-adjusted logistic regression of SDMA and ADMA with survival.

Model	SDMA	ADMA
Model 1 (age, sex)	2.145 (1.170–3.931) ***p* = 0.014**	97.596 (12.403–767.978) ***p* < 0.001**
Model 2 (age, sex, SOFA score)	0.994 (0.462–2.137) *P* = 0.987	8.744 (0.331–230.919) *P* = 0.194
Model 3 (age, sex, leukocyte count)	2.996 (1.007–8.913) ***p* = 0.048**	40.354 (1.331–1223.774) ***p* = 0.034**
Model 4 (age, sex, CRP)	3.682 (0.863–15.717) *p* = 0.078	60.311 (1.677–2168.506) ***p* = 0.025**
Model 5 (age, sex, PCT)	1.601 (0.266–9.651) *p* = 0.608	0.428 (0.001–259.801) *p* = 0.795
Model 7 (age, sex, eGFR)	4.433 (0.496–39.606) *p* = 0.187	45.498 (0.534–3874.736) *p* = 0.092

Significant values are in bold.

Data show Cox’ regression coefficients with their 95% confidence intervals and *p* values of the adjusted models.

ADMA, asymmetric dimethylarginine; CRP, C-reactive protein; eGFR, estimated glomerular filtration rate; PCT, pro-calcitonin; SDMA, symmetric dimethylarginine; SOFA, sequential organ failure assessment.

**Figure 3:**
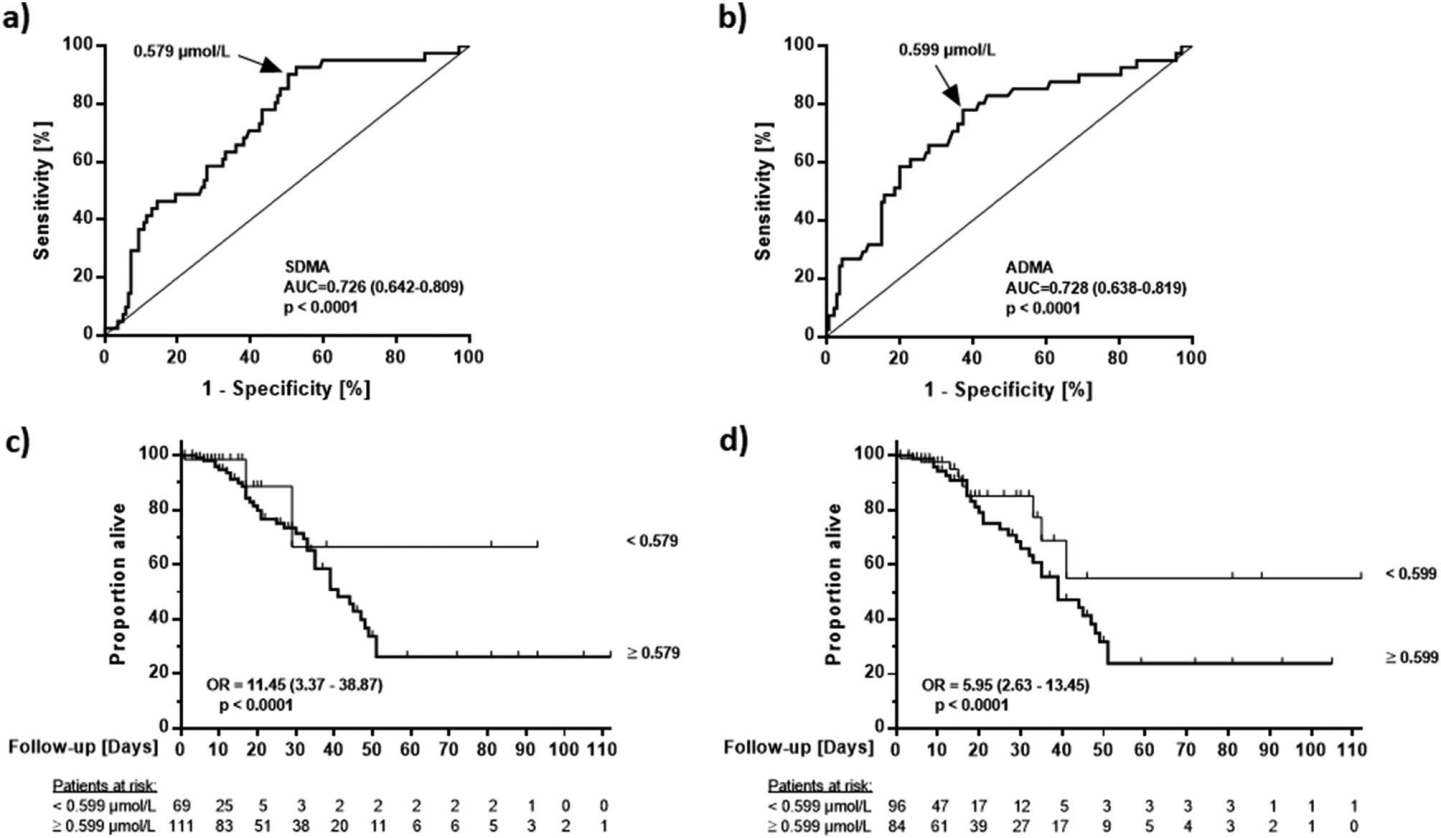
Receiver-operated curve (ROC) charts for SDMA (**a**) and ADMA (**b**), and Kaplan–Meier survival curves for SDMA (**c**) and ADMA (**d**). The serum concentration allowing optimal discrimination between Covid-19 survivors and non-survivors is marked by arrows in (**a**) and (**b**); survival curves were constructed by splitting data at these pre-determined concentrations. ADMA, asymmetric dimethylarginine; AUC, area under the curve; OR, odds ratio; SDMA, symmetric dimethylarginine.

In receiver-operated curve (ROC) analyses for each compound against mortality, SDMA had an AUC of 0.726 (0.642–0.809; *p* < 0.001), and ADMA had an AUC of 0.728 (0.638–0.819; *p* < 0.001) (Fig. 3a,b). The optimal ADMA cut-off concentration to discriminate between fatal cases and survivors was determined as 0.599 µmol/L (sensitivity, 0.780; specificity, 0.626), and the optimal cut-off for SDMA was determined as 0.579 µmol/L (sensitivity, 0.927; specificity, 0.475).

Using these cut-off values, we performed Kaplan–Meier analyses. Patients whose serum SDMA concentration or ADMA concentration was above the cut-off concentration had significantly higher mortality rates than those with serum SDMA or ADMA equal to or below the respective threshold concentration. The odds ratio for COVID-19-associated death in patients with SDMA > 0.579 µmol/L was 11.45 (3.37–38.87; *p* < 0.0001; Fig. 3c), and the odds-ratio in patients with ADMA > 0.599 µmol/L was 5.95 (2.63–13.45; *p* < 0.0001; Fig. 3d).

### Associations of combined ADMA and SDMA with mortality in COVID-19

**Figure 4:**
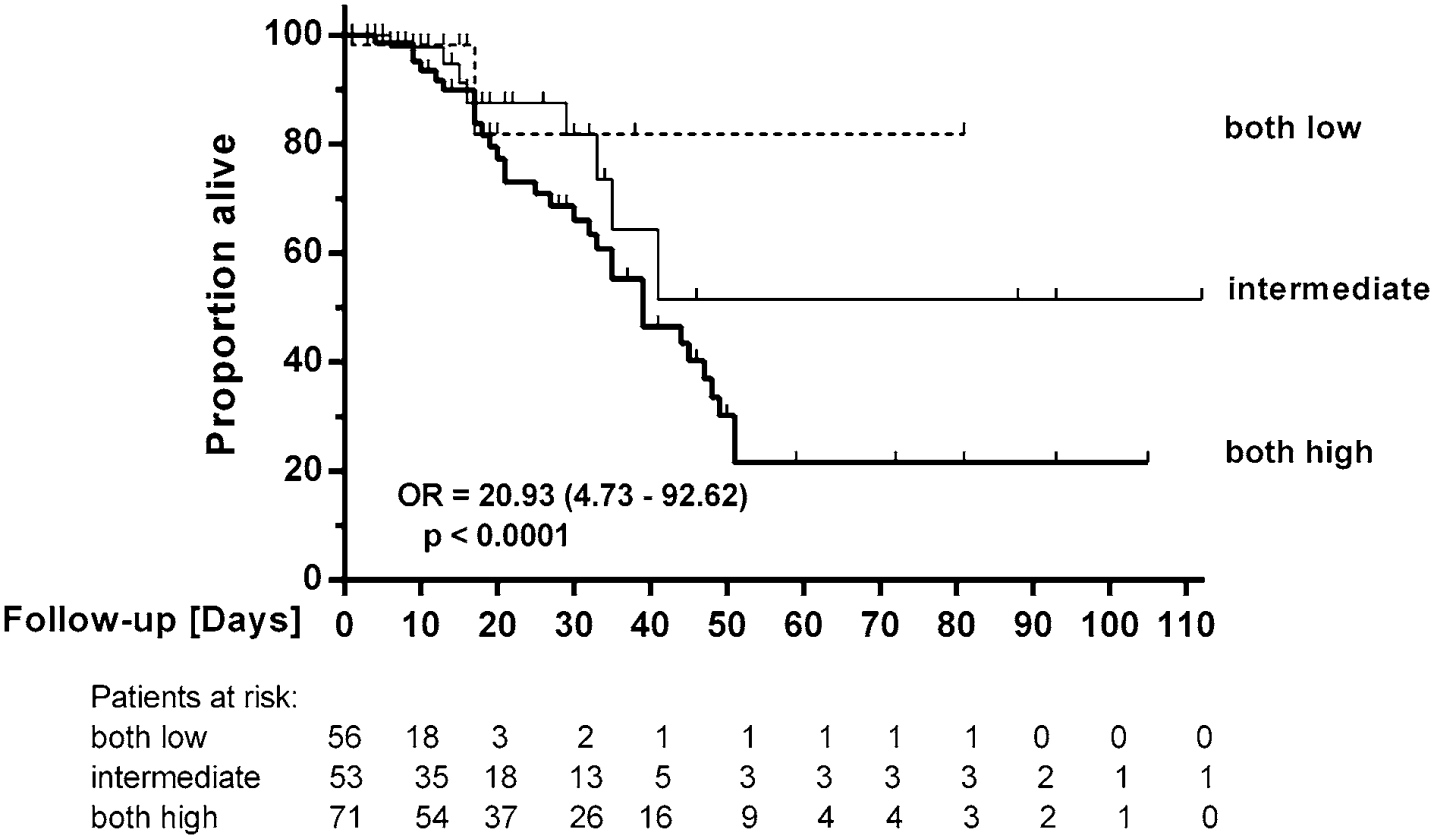
Kaplan–Meier survival curves for combined analysis of ADMA and SDMA serum concentrations. Both low means both biomarker concentrations below their respective cut-off levels from ROC analysis (see Fig. 2a,b); intermediate means that one biomarker is above and the other is below the respective cut-off level; both high indicates that both biomarkers were above their respective cut-off levels. OR, odds ratio.

**Figure 5:**
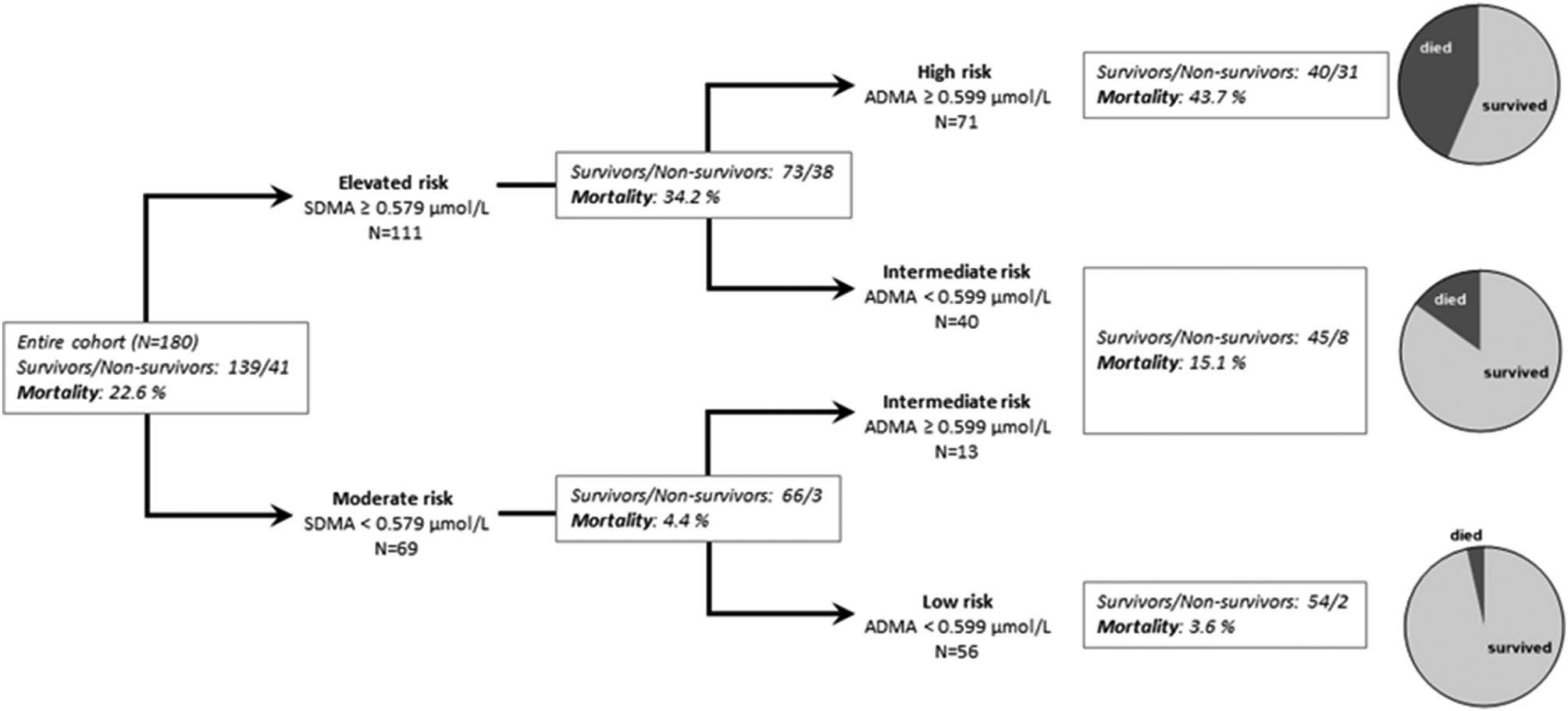
Decision tree analysis for assessing the risk of in-hospital mortality based on sequential analysis of SDMA and ADMA. Out of 180 patients, 41 died (22.6%). First decision step: Patients were identified as having elevated risk when SDMA levels were ≥ 0.579 μmol/L, and moderate risk when SDMA levels were < 0.579 µmol/L. Second decision step: Additional analysis of ADMA allowed identification of patients with high risk (SDMA ≥ 0.579 µmol/L and ADMA ≥ 0.599 µmol/L (mortality, 43.7%), intermediate risk (SDMA ≥ 0.579 µmol/L or ADMA ≥ 0.599 µmol/L (mortality, 15.1%), or low risk (SDMA < 0.579 µmol/L and ADMA < 0.599 µmol/L (mortality, 3.6%).

Next, we tested whether the combined analysis of SDMA and ADMA improved predictive power using a decision tree algorithm. Sequential measurements of SDMA and ADMA significantly enhanced the discrimination of mortality risk (Fig. 4). Patients with both, high SDMA and high ADMA concentrations had a high mortality risk of 43.7%, as compared to an intermediate risk of 15.1% in patients with either SDMA or ADMA elevated, and a low mortality risk of 3.6% in patients with both ADMA and SDMA low (Fig. 5). The odds ratio for patients in the intermediate risk group versus low risk group was 4.80 (1.03–23.76; *p* = 0.049); the odds ratio in the high versus low risk group was 20.93 (4.73–92.62; *p* < 0.0001). In ROC analyses, (ADMA + SDMA) and (ADMA x SDMA) resulted in AUCs of 0.734 (0.648–0.821) and 0.741 (0.654–0.828), respectively (both *p* < 0.0001) (Supplementary Figure 2).

### Improved prediction of disease severity with ADMA and SDMA compared to classical inflammatory laboratory markers

The well-established inflammatory laboratory markers, leukocyte cell count, C-reactive protein (CRP), and pro-calcitonin (PCT), as well as the SOFA score at admission were all significantly associated with mortality risk, with the relative risks for total mortality associated with each of these traditional markers lying in the range of 2–threefold (Table 4). Amongst combinations of these classical risk markers, SOFA and leukocyte count, SOFA and PCT, CRP and leukocyte count, and CRP and PCT produced the strongest increases in hazard ratio (Supplementary Figure 3a).

**Table 4:** ROC analyses for ADMA, SDMA, and traditional laboratory markers versus mortality.

	AUC	95% CI	Cut-off*	Sensitivity	Specificity	Hazard Ratio^#^	95% CI	*p*
ADMA	0.728	0.638–0.819	0.599	0.780	0.626	1.46	1.22–1.75	< 0.0001
SDMA	0.726	0.642–0.809	0.579	0.927	0.475	1.45	1.26–1.68	< 0.0001
(ADMA + SDMA)	0.734	0.648–0.821	1.411	0.756	0.655	1.48	1.23–1.79	< 0.0001
(ADMAxSDMA)	0.741	0.654–0.828	0.363	0.854	0.576	1.48	1.26–1.75	< 0.0001
SOFA score	0.846	0.774–0.918	4.5	0.656	0.875	2.68	1.60–4.50	< 0.0001
Leukocyte count	0.719	0.574–0.864	9.9	0.667	0.793	1.77	1.17–2.67	0.0008
CRP	0.777	0.641–0.912	118.5	0.737	0.746	3.27	1.41–7.62	< 0.0001
PCT	0.821	0.676–0.965	0.17	0.846	0.756	1.97	1.25–3.12	0.0002
D-dimers	0.609	0.477–0.740	n.d	n.d	n.d	n.d	n.d	n.s
Creatinine	0.659	0.486–0.832	n.d	n.d	n.d	n.d	n.d	n.s

*The optimal cut-off for discrimination of survivors and non-survivors was determined for each variable by applying the Youden procedure^27^. ^#^Hazard Ratios were calculated for comparison mortality risk of patients with risk marker levels above as compared to equal or below the cut-off value.

ADMA, asymmetric dimethylargin–ine; CRP, C-reactive protein; PCT, pro-calcitonin; SDMA, symmetric dimethylarginine; SOFA, sequential organ failure assessment.

We next tested the combination of traditional laboratory markers and SOFA with SDMA and ADMA (Table 5). SOFA score in combination with ADMA, SDMA, or their combination did not result in improved risk prediction (Supplementary Figure 3b), nor did leukocyte count or PCT (Supplementary Figure 3c,d). By contrast, the combination of CRP with ADMA alone, (ADMA + SDMA), and (ADMA x SDMA) resulted in the greatest increases in hazard ratio (Supplementary Figure 3e). COVID-19 patients with elevated CRP and elevated (ADMA x SDMA) had a hazard ratio for mortality of 10.0 (1.56–64.23), *p* < 0.0001.

**Table 5:** Predictive power of the SOFA score, C-reactive protein levels, and pro-calcitonin levels at admission for in-hospital mortality, when analyzed alone or in combination with SDMA or ADMA.

Parameter	Mortality [%] low/intermediate^a^/high	P*	Hazard Ratio for high versus low group	*p*^#^
SOFA Score	13.6/67.7		2.68 (1.60–4.50)	< 0.0001
SOFA Score + SDMA	0/23.9/70.4	< 0.0001	3.38 (1.89–6.04)	< 0.0001
SOFA Score + ADMA	6.4/27.5/73.9	< 0.0001	3.58 (1.79–7.16)	< 0.0001
Leukocytes	11.5/50.0		1.77 (1.72–2.67)	0.0008
Leukocytes + SDMA	0/28.3/61.1	< 0.0001	2.57 (1.44–4.59)	< 0.0001
Leukocytes + ADMA	7.1/33.3/60.0	0.0002	2.32 (1.24–4.35)	0.0003
C-reactive protein	12.7/73.3		3.27 (1.41–7.62)	< 0.0001
C-reactive protein + SDMA	0/27.6/78.6	< 0.0001	4.67 (1.71–12.73)	< 0.0001
C-reactive protein + ADMA	3.1/33.3/88.9	< 0.0001	8.72 (1.37–55.42)	< 0.0001
Pro-calcitonin	6.1/52.4		1.97 (1.25–3.12)	0.0002
Pro-calcitonin + SDMA	0/28.7/57.9	0.0002	2.38 (1.40–4.02)	0.0002
Pro-calcitonin + ADMA	0/43.9/61.5	0.0003	2.60 (1.31–5.17)	0.0002

^a^Mortality of the subgroups is given for the dichotomized SOFA score, C-reactive protein, and pro-calcitonin values when analyzed alone (no intermediate risk group), as well as for the highest risk group (traditional risk marker high plus SDMA/ADMA high), the intermediate risk group (traditional risk marker or SDMA/ADMA high), and lowest risk group (traditional risk marker plus SDMA/ADMA low).

*P denotes statistical significance level for trend across all risk groups.

^#^*p* denotes statistical significance level for the hazard ratio of the high versus low-risk groups.

ADMA, asymmetric dimethylarginine; SDMA, symmetric dimethylarginine; SOFA, sequential organ failure assessment.

### Time course of ADMA and SDMA during clinical treatment in COVID-19 survivors and non-survivors

**Figure 6:**
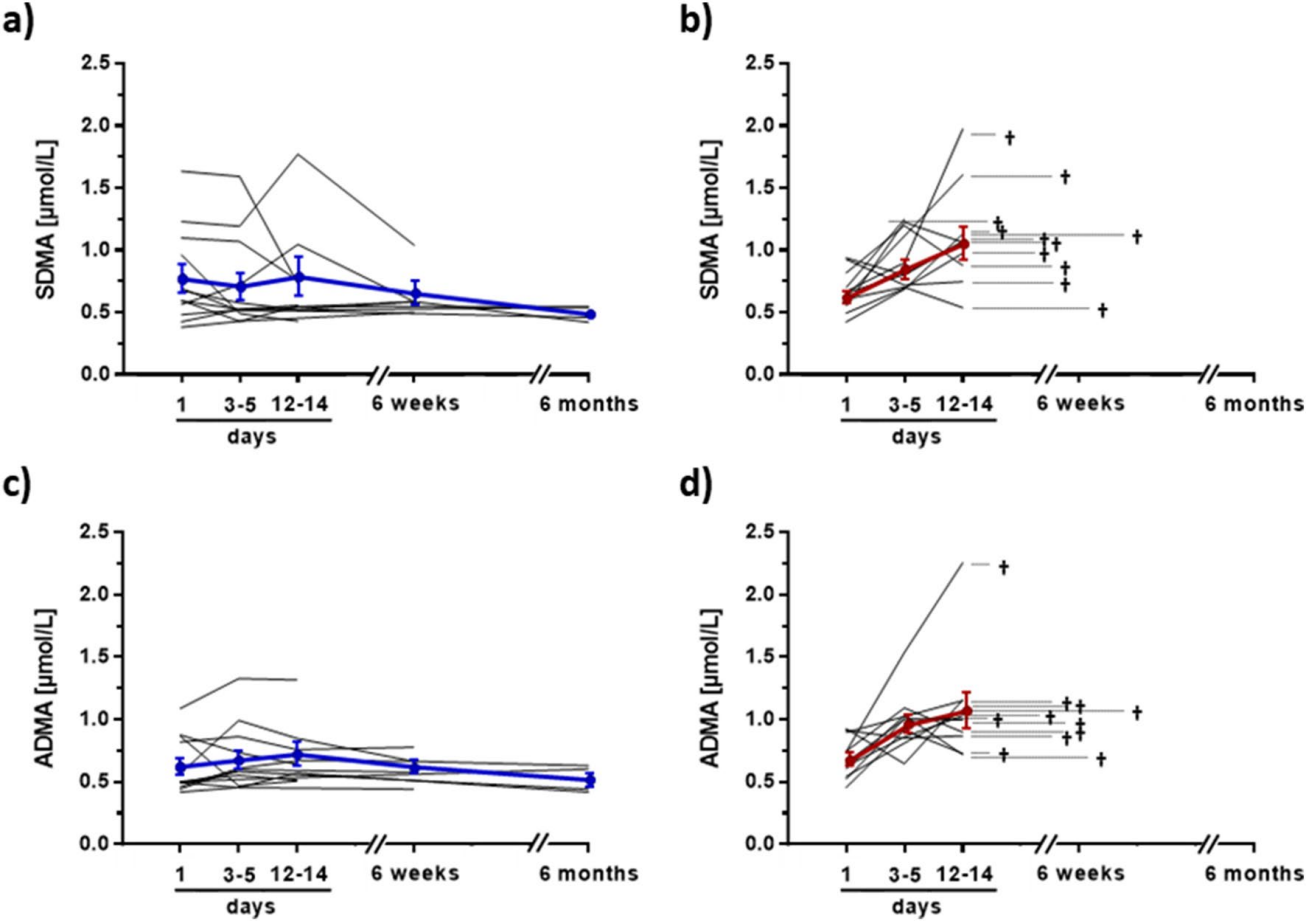
Time course of serum concentrations of SDMA and ADMA in Covid-19 patients who survived (**a**,**c**) or died (**b**,**d**) during hospitalization. The coloured lines indicate the groups’ means and standard deviations at each time point (blue, survivors, red, non-survivors). Dotted horizontal lines in plots (**b**) and (**d**) mark the time that elapsed until the day of death of COVID-19 non-survivors. ADMA, asymmetric dimethylarginine; SDMA, symmetric dimethylarginine.

Consecutive blood samples from 23 COVID-19 patients (12 survivors, 11 non-survivors) were available for analysis. The serum concentrations of SDMA and ADMA significantly increased in non-survivors during the first 14 days in hospital, i.e., in the period before their death. By contrast, both biomarkers showed stable concentrations over time in survivors (Fig. 6a–d). As a consequence, the L-arginine/ADMA ratio significantly decreased over time in non-survivors (Supplementary Figure 4a). The time course of the L-citrulline/L-arginine ratio showed no clear differentiation between survivors and non-survivors (Supplementary Figure 4b), whereas we observed a significant increase in L-ornithine/L-arginine ratio over time in non-survivors (Supplementary Figure 4c).

## Discussion

Our study provides evidence from a multicenter study that quantification of ADMA and SDMA in blood samples taken as early as possible after admission of COVID-19 patients to hospital contribute significantly to enhanced estimation of patients’ mortality risk during in-hospital treatment. Whilst each of the two biomarkers alone had limited prognostic value and was not superior to classical risk determinants in routine clinical chemistry and clinical scoring, addition of either ADMA or SDMA to such traditional risk predictors significantly improved the predictive power. Sequential analysis of both, ADMA and SDMA, allowed discrimination of a high risk, medium risk, and low risk group of COVID-19 patients with greatly different rates of in-hospital mortality. In addition, the time courses of ADMA and SDMA in repetitive blood samples during the first two weeks of hospitalization showed significant dichotomy between survivors and non-survivors, respectively.

The hospital mortality rate in the patient population included in this study was 20.7%, which compares to 29% in our pilot study^15^, 11% in the study by Sozio and co-workers^19^, and 22% in another study by Karacaer and co-workers^18^. Non-survivors in our study were significantly older than survivors, had lower systolic and diastolic blood pressure and higher systemic inflammatory markers, and required more intensive treatment including extracorporeal membrane oxygenation in a high percentage of patients. These differences in clinical characteristics were mirrored by differences in L-arginine-related biomarkers: As compared to controls, COVID-19 patients had higher L-ornithine concentrations, indicative of arginase activation, as well as higher L-citrulline concentrations, indicative of increased NOS enzymatic activity, whereas L-arginine concentrations were not significantly different. L-ornithine concentrations were also significantly elevated in non-survivors as compared to survivors, and the Orn/Arg Ratio significantly increased over time in non-survivors as opposed to survivors.

Most notably, however, the dimethylated L-arginine metabolites, ADMA and SDMA, were elevated in COVID-19 patients compared to controls; both were higher in non-survivors than in survivors, and they showed a further increase in non-survivors during the first two weeks of hospitalization. Taken together, these two biomarkers made it possible to significantly discriminate between COVID-19 patients with low, intermediate, and high mortality risk. Further, these two biomarkers significantly enhanced the predictive power of classical inflammatory markers. Thus, our study proves that ADMA and SDMA serum concentrations are suitable risk markers in severely diseased COVID-19 patients, and they corroborate our previous findings as well as those reported for small patient samples by other investigators. Using an untargeted metabolomics profiling approach in 27 COVID-19 patients compared to 36 healthy controls, Alboniga and co-workers reported significantly elevated ADMA and SDMA concentrations as well as decreased L-citrulline concentrations in COVID-19 patients^16^. Hasimi and colleagues compared 57 patients with moderately severe COVID-19 disease with 29 patients with severe COVID-19 and 21 healthy controls. They found significantly elevated ADMA in all COVID-19 patients as compared to controls, and a gradual increase in SDMA which was highest in patients with severe COVID-19^17^. In another study using a sandwich ELISA technique to measure ADMA, Karacaer and colleagues reported a higher ADMA increase in patients with severe COVID-19 than in those with a mild disease course^18^.

L-arginine metabolism is involved in numerous biochemical processes that play a role during systemic inflammation: L-arginine serves as a substrate for NO synthase (NOS), where the inducible isoform of NOS (iNOS) is involved in unspecific host defense^7^. At the same time, L-arginine is also the substrate for endothelial NOS, which generates NO by a much smaller catalytic rate and is an important physiological regulator of the homeostasis in vascular tone and vascular function^28^. From our measurements of L-arginine and L-citrulline, the by-product of NOS, we cannot deduct which isoform of NOS was responsible for the conversion of L-arginine to L-citrulline; therefore, this metabolic ratio remains a rough surrogate measure of total NOS activity—even more so as other biochemical pathways may also affect L-arginine and L-citrulline concentrations. However, the differences in metabolite concentrations between survivors and non-survivors that we noted in our study are suggestive of higher NOS activity and higher arginase activity in patients who died of COVID-19. Upregulation of inducible iNOS is commonly seen in sepsis patients, where excessive NO production contributes to low blood pressure which may lead to septic shock^29^. Interestingly, arterial blood pressure was lower in patients who died than in survivors within our multicenter cohort, supporting the hypothesis that iNOS induction may have contributed to their fatal outcome.

L-arginine can also be converted to L-ornithine by either of two isoforms of arginase^30^. The Orn/Arg Ratio serves as a metabolic surrogate marker of arginase activity; however, the metabolic flux of L-ornithine to, e.g., polyamines or collagen, also determines its levels. Whilst arginase-1 is highly expressed in hepatocytes, red blood cells, and immune cells, arginase-2 is a more ubiquitous enzyme that can be upregulated by inflammatory cytokines^30^. Therefore, we speculate that the systemic inflammation present in those COVID-19 patients that ultimately did not survive hospitalization may have contributed to higher arginase activity and, thus, higher Orn/Arg Ratio in our study.

The core result of our study is that ADMA and SDMA are elevated in hospitalized COVID-19 patients at the time of hospitalization. Although our study is limited by the fact that we were unable to include healthy controls of high age, the finding that the levels of ADMA and SDMA were even higher in patients with high risk of mortality and further increased in non-survivors as compared to survivors strongly suggest that the differences we measured between groups are meaningful. In the quest for more reliable risk predictors of COVID-19-associated mortality, our previous pilot study as well as a small number of other studies with small patient numbers suggested that ADMA and SDMA may be suitable for risk prediction in COVID-19. We confirm these prior observations here in a multicenter study design reporting that cut-off values of 0.599 µmol/L for ADMA and 0.579 µmol/L for SDMA are optimal discriminators of high- and low-risk COVID-19 patients during their hospitalization.

Our study is limited by the fact that the control group was matched by age and sex, but not for underlying health conditions. Thus, some of the difference in ADMA and SDMA concentrations between COVID-19 patients and healthy controls may have been due to the difference in mean age and the absence of chronic diseases like chronic lung disease, heart diseases, and diabetes mellitus in the control group. To this end, we performed a supplementary analysis restricted to 127 fully age-matched patients and controls, in which we found very closely similar differences in mean biomarker concentrations like in the complete dataset. Therefore, the known age-dependence of plasma ADMA and SDMA concentrations appears not to have had a major impact on the differences in biomarker levels in the present study. In addition, the major comparison in our study was between survivors and non-survivors among COVID-19 patients. There were some differences in the prevalence of co-morbidities between survivors and non-survivors as displayed in Table 1. However, the “net effect” of the co-morbidities on ADMA and SDMA levels is difficult to account for, as, for example, chronic lung disease, which is associated with high ADMA, was more prevalent in non-survivors, whilst cardiovascular disease and chronic kidney disease, which are also associated with high ADMA, were less prevalent in non-survivors.

The mechanism leading to upregulation of SDMA and ADMA concentrations may be related to systemic inflammation. We recently reported associations of these two biomarkers with inflammation in the population-based SHIP cohort^31^. In previous studies, we had found a strong interrelation between ADMA and CRP in predicting the progression of vascular intimal damage in hemodialysis patients^32^, and proposed that upregulation of DDAH activity during sepsis might counterbalance induction of iNOS activity^33^. Accordingly, in sepsis patients, we found that SDMA and ADMA predicted survival in a cohort of 120 ICU-treated patients with sepsis^9^. In the latter study, SDMA was correlated with pro-calcitonin and creatinine serum levels whilst ADMA was not. Both markers were correlated with lactate levels, which is suggestive of a relation to microcirculatory failure and tissue hypoxia. Finally, our previous study^19^ demonstrated that amongst COVID-19 patients classified into low, intermediate, and high risk groups by a machine-learning approach, ADMA was highest in the high-risk group and it was associated by absence of pulmonary vasodilation as analyzed in thoracic CT scan. These findings are in line with previous work by our group dissecting the molecular mechanisms of regulation of ADMA and SDMA in pulmonary hypoxia and the role of ADMA in modulating hypoxic pulmonary vasoconstriction^34^. Clearly, a mechanistic link of SARS-CoV-2 virus to ADMA and SDMA metabolism cannot be established from our clinical cohort study, but the existing data from the present study and the previous studies cited above clearly warrant further research into possible mechanisms. Systemic inflammation and pulmonary hypoxia might be two conditions worth being explored in this context.

In conclusion, our study strongly suggests that adding ADMA and SDMA as biomarkers to our portfolio of diagnostic assessment in hospitalized COVID-19 patients provides significant benefit for early discrimination of patients with a severe, life-threatening disease progression. This may help to better allocate resources to patients at high risk of in-hospital mortality.

## Supplementary Information

Supplementary Information.

## Data Availability

All data generated or analysed during this study are included in this published article and its supplementary information files.
