# Author Correction: A multicenter study of asymmetric and symmetric dimethylarginine as predictors of mortality risk in hospitalized COVID-19 patients

**DOI:** 10.1038/s41598-025-99224-0

**Published:** 2025-04-29

**Authors:** Juliane Hannemann, Anne Zink, Yoana Mileva, Paul Balfanz, Edgar Dahl, Sonja Volland, Thomas Illig, Edzard Schwedhelm, Florian Kurth, Alexandra Stege, Martin Aepfelbacher, Armin Hofmann, Rainer Böger

**Affiliations:** 1 Institute of Clinical Pharmacology and Toxicology, University Medical Center Hamburg-Eppendorf, Hamburg, Germany; 2 Department of Cardiology, Angiology and Intensive Care Medicine, Medical Clinic I, University Hospital Aachen, Aachen, Germany; 3 Department of Physiology, Henri Mondor Hospital, FHU-SENEC, INSERM U955, Université Paris-Est Créteil (UPEC), AP-HP, Créteil, France; 4 Institute of Pathology and Central Biobank, University Hospital Aachen, Aachen, Germany; 5 Hannover Unifed Biobank, Medizinische Hochschule Hannover, Hannover, Germany; 6 German Centre for Cardiovascular Research (DZHK), Partner Site Hamburg/Kiel/Lübeck, Hamburg, Germany; 7 Department of Infectious Diseases and Critical Care Medicine, Charité Universitätsmedizin Berlin, Berlin, Germany; 8 Central Biobank Charité, Charité Universitätsmedizin Berlin, Berlin, Germany; 9 Institute of Medical Microbiology, Virology and Hygiene, University Medical Center Hamburg-Eppendorf, Hamburg, Germany

Correction to: *Scientific Reports* 10.1038/s41598-024-66288-3, published online 08 July 2024

In the original version of this Article Paul Balfanz was incorrectly affiliated with ‘Institute of Pathology and Central Biobank, University Hospital Aachen, Aachen, Germany.’ Their correct affiliations are listed below.

Department of Cardiology, Angiology and Intensive Care Medicine, Medical Clinic I, University Hospital Aachen, Aachen, Germany.

Department of Physiology, Henri Mondor Hospital, FHU-SENEC, INSERM U955, Université Paris-Est Créteil (UPEC), AP-HP, Créteil, France.

Additionally, Edgar Dahl was incorrectly affiliated with ‘Hannover Unified Biobank, Medizinische Hochschule Hannover, Hannover, Germany.’ Their correct affiliation is listed below.

Institute of Pathology and Central Biobank, University Hospital Aachen, Aachen, Germany.

Lastly, Sonja Volland and Thomas Illig were incorrectly affiliated with ‘Department of Physiology, Henri Mondor Hospital, FHU-SENEC, INSERM U955, Université Paris-Est Créteil (UPEC), AP-HP, Créteil, France’. Their correct affiliation is listed below.

Hannover Unified Biobank, Medizinische Hochschule Hannover, Hannover, Germany.

The original Article and accompanying Supplementary Information file have been corrected.

