# Supplementary material for: A multicenter study of asymmetric and symmetric dimethylarginine as predictors of mortality risk in hospitalized COVID-19 patients
Source: Sci Rep. 2024 Jul 8;14:15739. doi: 10.1038/s41598-024-66288-3 (PMC11231343; doi:10.1038/s41598-024-66288-3)
Supplement: Supplementary file 1 — Supplementary Information. [file 41598_2024_66288_MOESM1_ESM.pdf]

A multicenter study of asymmetric and symmetric dimethylarginine as predictors of mortality risk in hospitalized COVID-19 patients.

Supplementary Materials

Juliane Hannemann[1], Anne Zink[1], Yoana Mileva[1], Paul Balfanz[2, 3], Edgar Dahl[4], Sonja Volland[5], Thomas Illig[5], Edzard Schwedhelm[1,6], Florian Kurth[7], Alexandra Stege[8], Martin Aepfelbacher[9], Armin Hoffmann[9], Rainer Böger[1,6]*

[1] Institute of Clinical Pharmacology and Toxicology, University Medical Center Hamburg-Eppendorf, Hamburg, Germany; [2] Department of Cardiology, Angiology and Intensive Care Medicine, Medical Clinic I, University Hospital Aachen, Germany; [3] INSERM U955, Université Paris-Est Créteil (UPEC), AP-HP, Department of Physiology, Henri Mondor Hospital, FHU-SENEC, Créteil, France; [4] Institute of Pathology and Central Biobank, University Hospital Aachen, Germany; [5] Hannover Unified Biobank, Medizinische Hochschule Hannover, Germany; [6] German Centre for Cardiovascular Research (DZHK), partner site Hamburg/Kiel/Lübeck, Germany; [7] Department of Infectious Diseases and Pulmonary Medicine, Charité Universitätsmedizin Berlin, Germany; [8] Central Biobank Charité, Charité Universitätsmedizin Berlin, Germany; [9] Institute of Medical Microbiology, Virology and Hygiene, University Medical Center Hamburg-Eppendorf, Hamburg, Germany

* Corresponding author:
Prof. Dr. Rainer Böger;
Phone: +49-40-7410-59759
Fax: +49-40-7410-59757
Email: boeger@uke.de

Supplementary Tables

Supplementary Table 1.

Biomarker concentrations at baseline in fully age- and sex-matched COVID-19 patients and controls.

	**Covid-19 patients**	**Controls**	**p**
N	127	127	
Sex (m / f)	83 / 44	83 / 44	n.s.
Age (years)	55.1 ± 12.2	54.9 ± 11.9	n.s.
L-Arginine	117.1 ± 41.2	100.3 ± 25.7	n.s.
L-Ornithine	109.6 ± 37.1	66.7 ± 18.4	**< 0.0001**
L-Citrulline	21.3 ± 9.7	35.6 ± 8.7	**< 0.0001**
ADMA	0.613 ± 0.200	0.480 ± 0.098	**< 0.0001**
SDMA	0.756 ± 0.467	0.519 ± 0.094	**< 0.0001**
Arg/ADMA Ratio	199.9 ± 61.3	212.5 ± 50.8	n.s.
Orn/Arg Ratio	1.034 ± 0.565	0.683 ± 0.177	**< 0.0001**
Cit /Arg Ratio	0.198 ± 0.104	0.370 ± 0.102	**< 0.0001**

Supplementary Figures

Supplementary Figure 1.

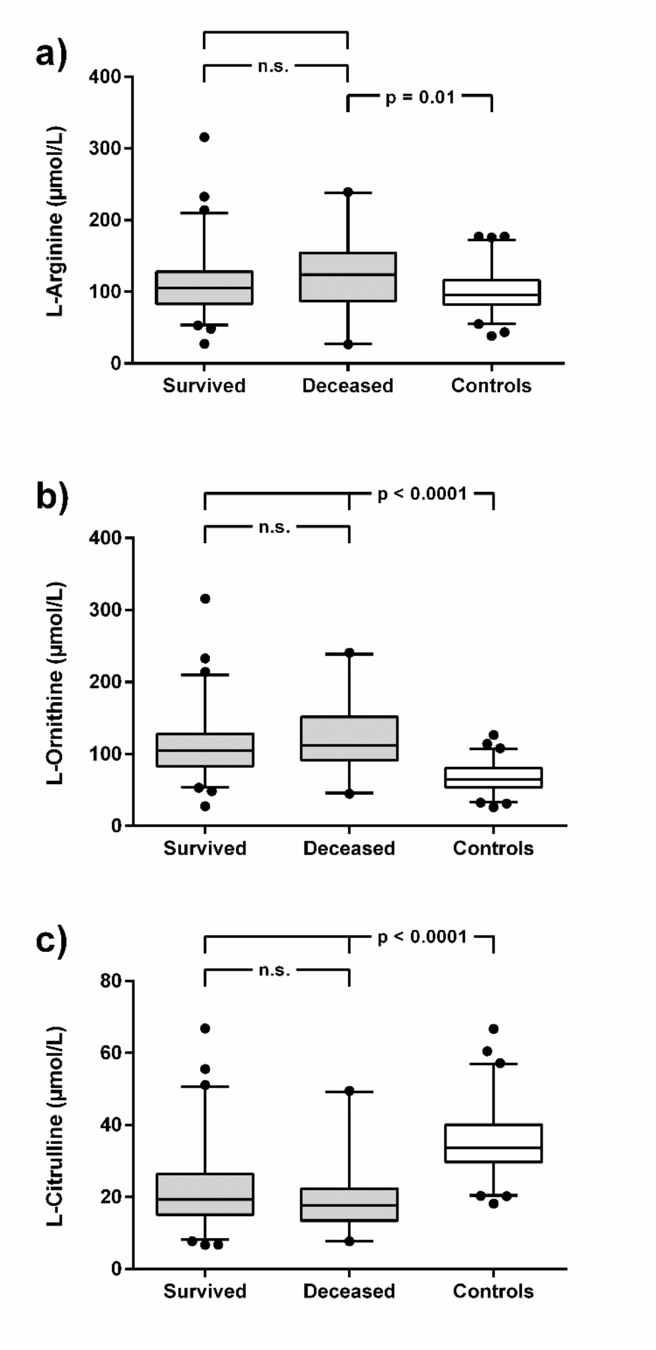

Box plots showing the plasma concentrations of L-arginine (**a**), L-ornithine (**b**), and L-citrulline (**c**) in hospitalized Covid-19 patients who survived or died during in-hospital treatment, as compared to age- and sex-matched healthy controls. Boxes show the median and interquartile range of the data, with whiskers representing the 2.5th to 97.5th percentiles; data points outside of this distribution are plotted individually. Statistical significances were calculated by one-way ANOVA followed by Tukey's multiple comparisons test.

Supplementary Figure 2.

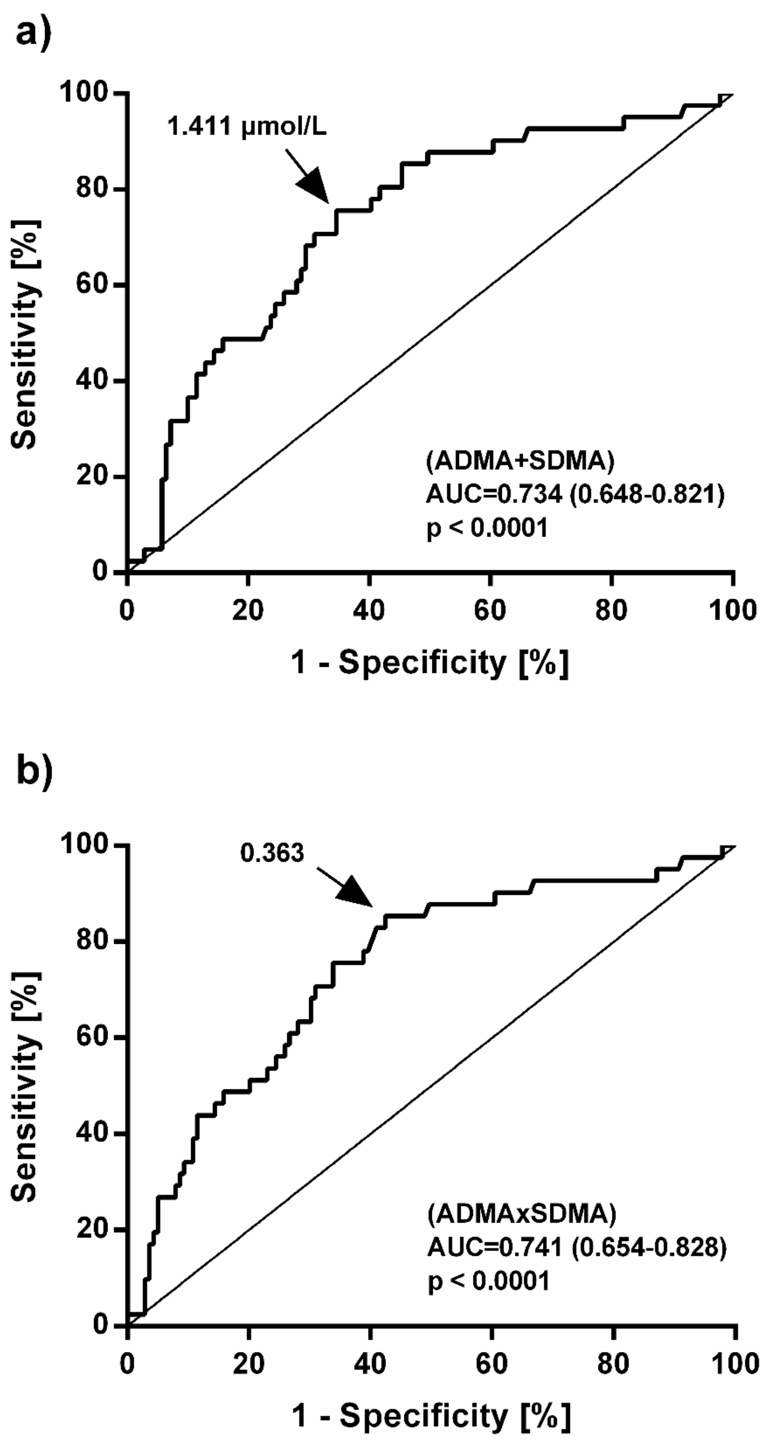

Receiver-operated curve (ROC) charts for the combined biomarker variables (ADMA + SDMA) (**a**) and (ADMA x SDMA) (**b**). The plasma concentration allowing optimal discrimination between Covid-19 survivors and non-survivors is marked by arrows. Abbreviations: ADMA, asymmetric dimethylarginine; AUC, area under the curve; OR, odds ratio; SDMA, symmetric dimethylarginine.

Supplementary Figure 3.

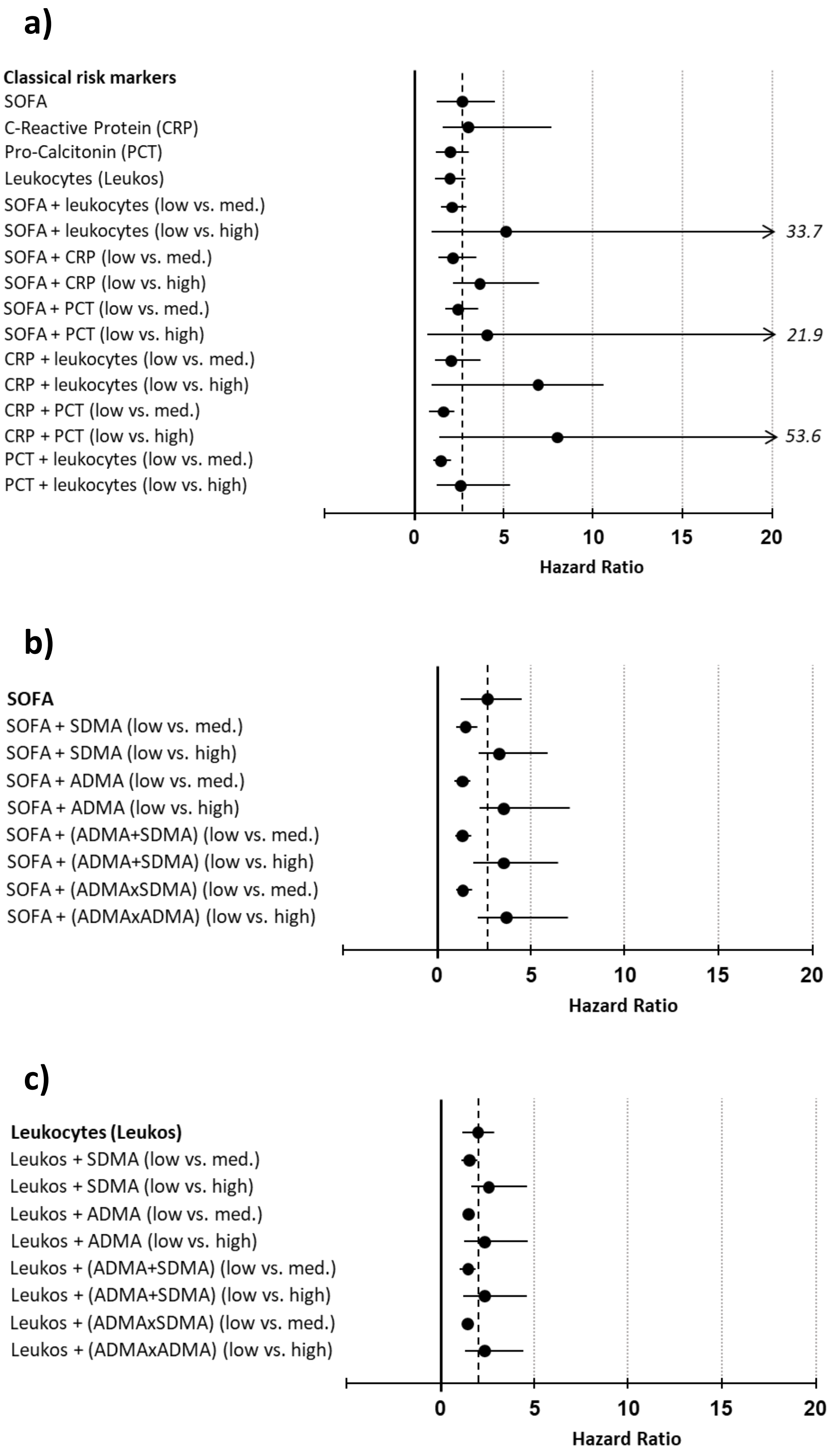

Supplementary Figure 3 (cont'd).

d)

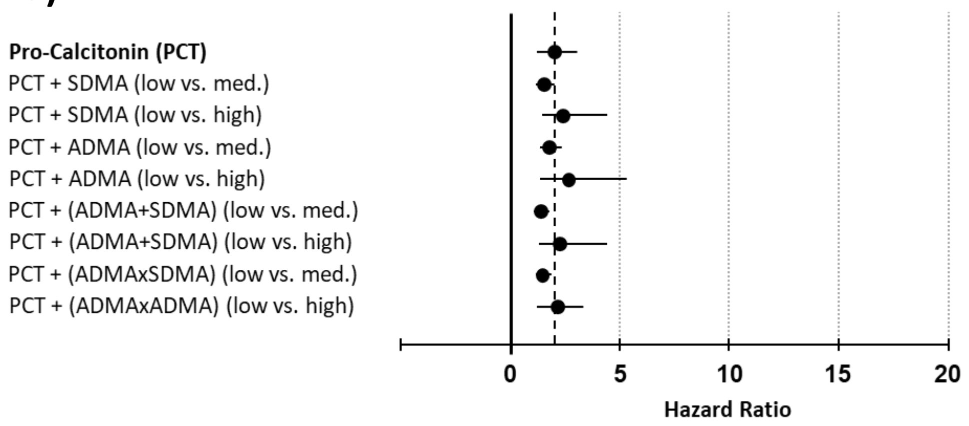

e)

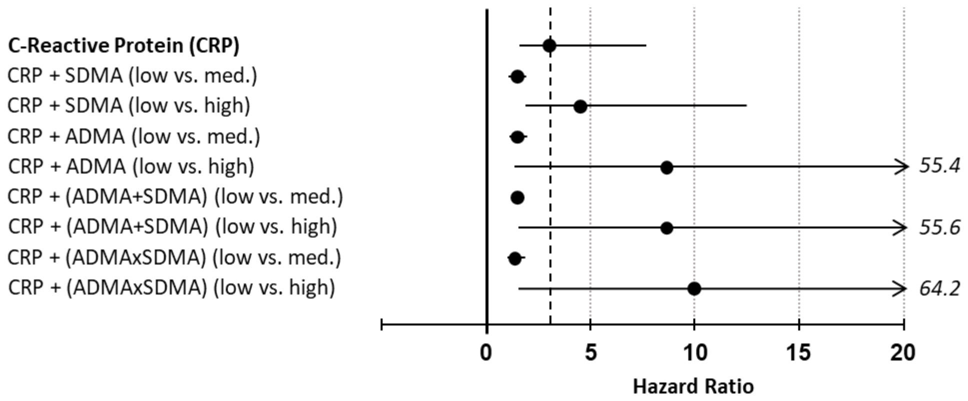

Hazard ratios for mortality of hospitalized Covid-19 patients. Data shown are hazard ratios with 95 % confidence intervals for classical risk markers (SOFA score, leukocyte cell count, pro-calcitonin, and C-reactive protein. In a, hazard ratios for these classical risk markers are shown either alone or for combinations amongst them. In b to e, hazard ratios are shown for each classical risk marker alone and in combination with SDMA, ADMA, (ADMA + SDMA) and (ADMA x SDMA). The upper limit of the 95 % confidence interval is plotted numerically if the scale of the x-axis is exceeded.

Supplementary Figure 4.

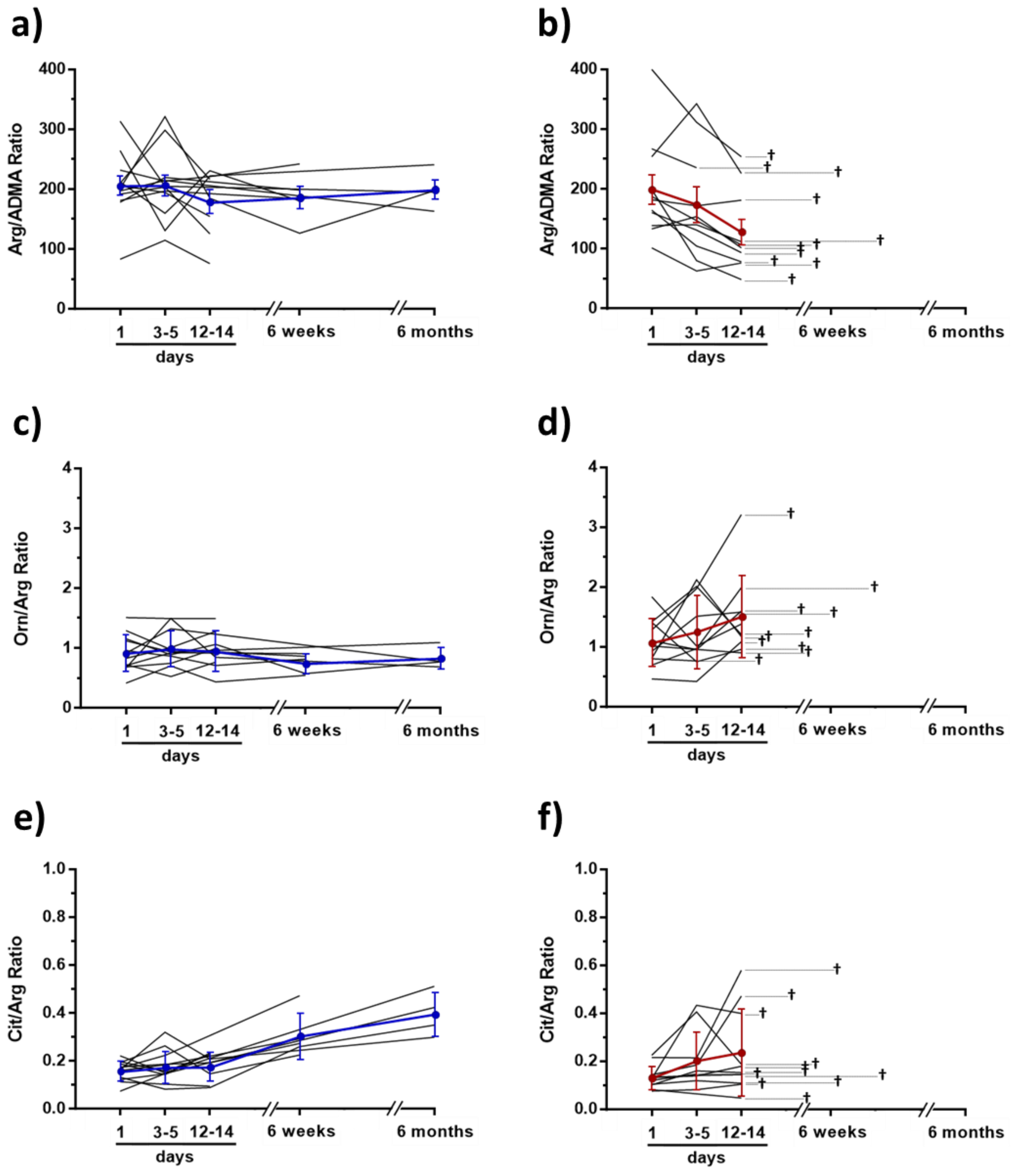

Time course of the plasma L-arginine/ADMA ratio (a and b), L-ornithine/L-arginine ratio (c and d), and L-citrulline/L-arginine ratio (e and f) in Covid-19 patients who survived (**a, c,** and **e**) or died (**b, d,** and **f**) during in-hospital treatment. The coloured lines indicate the groups' means and standard deviations at each time point (blue, survivors, red, non-survivors). Dotted lines in plots **b, d,** and **f** mark the time that elapsed until the day of death of Covid-19 non-survivors. Abbreviations: ADMA, asymmetric dimethylarginine; Arg, l-arginine; Cit, L-citrulline; Orn, L-ornithine.
